# Saccadic and manual response time data on inhibition of return during and after a visual search

**DOI:** 10.1016/j.dib.2021.107565

**Published:** 2021-11-12

**Authors:** Margit Höfler, Sebastian A. Bauch, Katrin Liebergesell, Iain D. Gilchrist, Anja Ischebeck, Christof Körner

**Affiliations:** a Department for Clinical Neurosciences and Preventive Medicine, Danube University Krems, Austria; b Institute of Psychology, University of Graz, Austria; c School of Psychological Science, University of Bristol, UK

**Keywords:** Visual search, Inhibition of return, Eye tracking, Saccadic responses, Manual responses

## Abstract

In the present paper we present a dataset that provides data of two experiments in which we investigated the presence of Inhibition of Return (IOR) during and after a visual search. Participants either had to saccade (Experiment 1 and 2) or make a manual response (Experiment 2) to a probe during a visual search task (searching for a target letter among a set of distractors) or immediately after its completion. The data consist of the unprocessed raw data and one csv-file of the processed eye tracking data on eight (Experiment 1) and 18 (Experiment 2) participants, respectively. In total, we obtained 5,116 trials in Experiment 1 and 18,424 in Experiment 2. The data set is stored at the repository DOOR hosted by the University of Krems (https://door.donau-uni.ac.at/view/o:1014). Detailed information about the experiments and the interpretation of the data can be found in the paper “Post-search IOR: Searching for inhibition of return after search” (Höfler et al., 2019) [1].

**Specifications Table**

**Table:** 

Subject	Experimental and Cognitive Psychology
Specific subject area	Experimental psychology, Eye tracking, Attention, Inhibition of Return
Type of data	edf files csv file
How data were acquired	Eye tracker (Eyelink2 and Eyelink1000; SR Research, Canada)
Data format	Raw Filtered
Parameters for data collection	In two eye-tracking experiments, participants searched a letter display and one letter was probed (flicker probe) during or immediately after the end of the search. The probed item was previously inspected (old probe) or not (new probe). Response latencies to the probe (saccadic in Experiment 1 and saccadic vs. manual in Experiment 2) were analyzed.
Description of data collection	Participants sat in a dark and soundproof room. The search displays were presented on a 21-in. CRT-monitor with a refresh rate of 100 Hz and a resolution of 1152 × 864 pixels. Viewing distance was approximately 63 cm. To minimize head movements participants had to rest their head on a chin rest. Manual responses were collected with a gamepad. Eye tracking data were collected from the eye which produced the better spatial resolution during the calibration phase (typically better than 0.31°).
Data source location	Institution: University of Graz, Institute of Psychology City/Town/Region: Graz Country: Austria
Data accessibility	Data are provided at the repository of the Danube University Krems, Austria. Repository name: DOOR (Repository of the Danube University Krems) Data identification number (data set): https://doi.org/10.48341/g72j-sk41 Direct URL to data collection (https://door.donau-uni.ac.at/view/o:1014) [2]
Related research article	Höfler, M., Liebergesell, K., Gilchrist, I. D., Bauch S. A., Ischebeck, A., & Körner, C. (2019). Post-search IOR: Searching for Inhibition of Return after search. *Acta Psychologica, 197*, 32–38. 10.1016/j.actpsy.2019.04.017[1]

**Value of the Data**

• The dataset includes data of two eye-tracking experiments (8 and 18 participants) on a visual search task in which inhibition of return is investigated.
• Data are useful for reanalysis regarding, for instance, a possible change of the magnitude of saccadic and manual IOR over the course of trials and or how the measurement of IOR with probes affects the visual search.
• The dataset does not only include the data of the experiments presented and discussed in Höfler et al. [1] but also further data such as response times to the search, incorrectly solved trials etc.

## 1 Data Description

The dataset is located at the repository DOOR of the Danube University Krems (https://door.donau-uni.ac.at/view/o:1014) [2] and is provided in .csv format in order to make it accessible for scholars. The doi-number of the data set is https://doi.org/10.48341/g72j-sk41. The data in this csv file were generated from the raw data files of the eye tracking experiments which are also located at the same repository address. The raw data files (edf-files that reflect the standard file format of SR-Research) were converted to ascii-Files via the EDF2ASC-converter provided by SR-Research and processed/filtered (see below) via Matlab and R-scripts. The resulting csv-file includes all variables analyzed in Höfler et al. [1] and further variables related to the search task. All variables provided in the data set are described in Table 1.

**Table 1:** Description of variables in the dataset.

Variable Name	Description
experiment	Data of Experiment in Höfler et al. [1] 1 = Experiment 1, 2 = Experiment 2
valid_trial	Indicates whether this trial was included in the analysis 0 = no, 1 = yes
code	Subject code (Experiment 1: string; Experiment 2: string/numeric)
search	Number of searches in the same display within a trial (numeric) 1 = first search, 2 = second search
block	Block of trials (numeric) 1–8 in Experiment 1; 1–4 (manual response) and 1–8 (saccadic response) in Experiment 2
trial	Trial number within block (numeric) 1–80 in Experiment 1; 1–86 in Experiment 2
response_cond	Indicates whether the response was manual or saccadic (numeric) 0 = saccadic, 1 = manual
target_cond	Indicates whether the target was absent or present in the search (numeric) 0 = absent, 1 = present
search_solved	Indicates whether the respective search (1 or 2) was solved correctly (numeric) 0 = incorrect; 1 = correct
trial_solved	Indicates whether both searches are solved correctly (numeric) 0 = one or both incorrect; 1 = both correct
rtime	Response time (ms) in respective search from search display onset until button press (numeric)
n_fix	Number of fixations in the respective search from Search display until button press (numeric)
target	Unique identity of the search target (numeric; 0–16)
tar_coord_x	x-coordinate of search target in pixel (numeric; 0 if target was absent)
tar_coord_y	*y*-coordinate of search target in pixel (numeric; 0 if target was absent)
probe_time	Indicates the onset of the probe (numeric) 0 = across searches, 1 = post search
probe_type	Indicates whether probe was “old”, “new” or not delivered (numeric) 0 = old probe, 1 = new probe, -1 = not delivered
probed_item	Identity of the probed items in the search (numeric; 0–16)
p_coord_x	*X* coordinate of the probed item in pixel
p_coord_y	*Y* coordinate of the probed item in pixel
rand_probe	Indicates whether an appropriate probe item was found that fits the criteria for probe presentation (see method section in [1]) 0 = yes, 1 = no
sacc1_to_probe	Indicates whether the first saccade after probe onset targeted the probed item (numeric) 1= yes, 0 = no
probe_onset_time	Indicates the internal time stamp of probe onset (in ms) during recording (numeric)
response_to_probe	Indicates the internal time stamp of the response to probe onset (in ms) during recording (numeric) (start of the saccade to the probe / button press)
latency	Response latency to probe (in ms) from probe onset until the start of the respective saccade to the probed item OR button press (numeric)
i*n*	Unique identification number of each of the 15 search items *n* = 1 to 15 presented in the display (numeric; 0 to 16)
i*n*_*x*_coord; i*n*_*y*_coord	Coordinates (*x*/*y*) of *n* = 1 to 15 items presented in the display in pixel (numeric)

## 2 Experimental Design, Materials and Methods

Eight naïve participants (two female, *M* = 24.4, range from 21 to 29 years) took part in Experiment 1; 18 new participants (11 female; *M* = 23.3 years; range from 20 to 29 years) in Experiment 2. The study was approved by the local ethics committee. Additionally, all gave informed consent and had normal or corrected-to-normal vision (contact lenses). All experimental manipulations in both experiments were made within subjects.

Detailed Method of Experiment 1: In each trial, participants were asked to search for a target letter during each of the two consecutive searches in the display consisting of 15 letters. The letters were presented in white Arial font (bold) on a black background and sampled randomly from a set of 17 letters of the Roman alphabet (the letters *B, C, D, J, N, Q, W, X*, and *Y* were omitted). The two remaining letters were used as targets in case of target-absent searches. The letters subtended approximately 0.32° and were surrounded by a circle (0.18° thick). The diameter of an item (letter and circle) was 0.9°. The 15 items were placed on the intersections of an imaginary 6 × 6 grid with a deviation within ± 0.23° both in horizontal and vertical direction on the display. The size of a grid cell was 3.6°.

At the beginning of each trial, participants were instructed to fixate a fixation disc for a drift correction and the experimenter started the trial manually when the fixation was registered. Afterwards, a placeholder display was presented for 500 ms. The placeholder display was identical to the upcoming search display except that each letter was replaced by the hash symbol (#). After that, the search display appeared and the first target letter was announced through loudspeakers. This target was present on half of the trials, and participants gave a manual absent/present response on a two-button response box. In order to measure IOR across the searches, one item was probed 300 ms after the start of the second search (i.e., after the first search was completed); to measure IOR post search, another item was probed 300 ms after the end of the second search. When an item was probed, it flickered, and the outer circle of the item changed its size briefly. The probed item was either an “old probe”; i.e., had either been within the last four item fixations in Search 1 (for across probes) or in Search 2 (for post-search probes) or was a “new probe”; i.e., the probed item had not been fixated during Search 1 (across probe) or during Search 2 (post-search probe). The participants were instructed to immediately saccade to this probe and continue the search. The distance between current fixated item and the probed items was held constant with about 10.8°. Saccadic latencies, i.e., the time between the onset of the probe and the start of the corresponding saccade to the probe, were used as the main dependent variable. Each participant completed eight blocks, divided into two or three sessions of two to four blocks on different days, with 80 trials each. 16 trials in each block were catch trials in which only one probe (either across the search or post-search) or no probe appeared. In total, 5120 trials were collected, four of which were lost due to technical problems. Hence, 5116 trials are available.

Detailed method of Experiment 2: Design, stimuli and procedure were the same as in the Experiment 1 except that participants were asked to search the 15-letter display only once and that one item was probed while the search was still ongoing (within-search probe) and immediately after the end of the search (post-search probe). The within-search probe was presented randomly after the fifth to ninth fixation of the participant during the search while the post-search probe was presented, similar to Experiment 1300 ms after the end of the search. Again, the probe was either recently inspected during search (old probe) or not (new probe). In Experiment 2, participants were either asked to saccade to the probe (eight blocks of trials) or press a button (four blocks of trials) once it appeared. Each block consisted of 86 trials including 18 catch trials. Participants were allowed to have short breaks of several minutes between blocks. In total, 18,576 trials were collected; 152 trials were lost due to technical problems, leaving in 18,424 trials.

Eye tracking: To collect the data, an EyeLink 2 eye tracker (SR Research, Ontario, Canada) with a sampling rate of 500 Hz was used in Experiment 1, and an Eyelink 1000+ eye tracker (SR Research, Ontario, Canada) with a sampling rate of 1000 Hz in Experiment 2. Velocity threshold for saccade detection was set to 35°/s and acceleration threshold to 9500°/s2 in both experiments. Data were collected from the eye which produced the better spatial resolution during the calibration phase (typically better than 0.31°). In Experiment 1, the search displays were presented on a 21-in. CRT-monitor with a refresh rate of 100 Hz. Viewing distance was approximately 63 cm. In Experiment 2, the stimuli were also presented on a 21 in. monitor, but with a refresh rate of 85 Hz. Viewing distance was approximately 75 cm. To minimize head movements participants had to rest their head on a chin rest in both experiments. Manual responses were collected with a gamepad.

## Ethics Statement

Before each experiment, participants gave informed consent. The described experiments were approved by the ethics committee of the University of Graz (GZ. 39/9/63 ex 2011/12 and GZ:39/62/63 ex 2016/17).

## CRediT authorship contribution statement

**Margit Höfler:** Conceptualization, Methodology, Software, Funding acquisition, Writing – original draft. **Sebastian A. Bauch:** Conceptualization, Methodology, Data curation, Writing – review & editing. **Katrin Liebergesell:** Conceptualization, Methodology, Investigation. **Iain D. Gilchrist:** Conceptualization, Methodology. **Anja Ischebeck:** Writing – review & editing. **Christof Körner:** Conceptualization, Methodology, Writing – review & editing.

## Declaration of Competing Interest

The authors declare that they have no known competing financial interests or personal relationships which have or could be perceived to have influenced the work reported in this article.
